# Facilitators and barriers to implementing a specialized care unit for persons with cognitive impairment in an acute geriatric hospital: a process evaluation

**DOI:** 10.1186/s12877-023-04612-8

**Published:** 2024-01-06

**Authors:** Laura Adlbrecht, Melanie Karrer, Nicole Helfenberger, Eva Ziegler, Adelheid Zeller

**Affiliations:** 1 Competence Center Dementia Care, Department of Health, Institute of Applied Nursing Sciences, Eastern Switzerland University of Applied Sciences, Rosenbergstrasse 59, 9000 St.Gallen, Switzerland; 2 Geriatrische Klinik St. Gallen AG, Rorschacher Strasse 94, St. Gallen, 9000 Switzerland; 3 University of Applied Sciences for Health Professions Upper Austria, Semmelweisstraße 34/D, Linz, 4020 Austria

**Keywords:** Dementia, Delirium, Cognitive impairment, Acute hospital, Acute care, Implementation science, Practice development, Process evaluation, Action research

## Abstract

**Background:**

Implementing dementia care interventions in an acute hospital poses multiple challenges. To understand factors influencing the implementation, in-depth knowledge about specific facilitators and barriers is necessary. The aim of this study was to identify facilitators and barriers to implementing an interprofessional, multicomponent intervention of a specialized unit for persons with cognitive impairment in an acute geriatric hospital.

**Methods:**

We conducted a process evaluation as part of a participatory action research study. For data collection, semi-structured individual interviews with fifteen professionals involved in the implementation of the specialized unit. We further conducted two focus groups with twelve professionals working on other units of the geriatric hospital. We performed a qualitative content analysis following Kuckartz’s content-structuring analysis scheme.

**Results:**

We identified the following barriers to implementing the specialized unit: uncontrollable contextual changes (e.g., COVID-19 pandemic), staff turnover in key functions, high fluctuation in the nursing team, traditional work culture, entrenched structures, inflexible and efficiency-oriented processes, monoprofessional attitude, neglect of project-related communication, and fragmentation of interprofessional cooperation. An established culture of interprofessionalism, an interprofessionally composed project group, cooperation with a research partner, as well as the project groups’ motivation and competence of managing change facilitated the implementation.

**Conclusions:**

The implementation faced numerous barriers that can be described using the key constructs of the i-PARIHS framework: context, recipients, innovation, and facilitation. Overcoming these barriers requires an organizational development approach, extended project duration and increased process orientation. Furthermore, strategically planned, precise and ongoing communication towards all persons involved seems crucial. Differences between the work cultures of the professions involved deserve particular attention with regard to project-related roles and processes.

**Supplementary Information:**

The online version contains supplementary material available at 10.1186/s12877-023-04612-8.

## Background

Persons with dementia have a significantly higher risk for hospital admission due to an acute physical illness, compared to same-aged persons without cognitive impairment [1]. The prevalence of persons with dementia in the acute hospital setting is estimated at 20-25% [2, 3]. Since 56% of dementia cases are not diagnosed or recognized by healthcare staff, prevalence may even be higher [4].

Due to strict routines and inflexible, entrenched processes, it is difficult to meet the needs of persons with dementia in an acute hospital [5–8]. A qualitative evidence-synthesis addressing the experiences of persons with dementia in hospitals shows that the busyness in acute hospitals and the missing privacy can result in an overstimulation of persons with dementia [9]. Simultaneously, persons with dementia feel “lost and bored” [10] due to a lack of meaningful activities or social interactions [9, 10]. It was shown that persons with dementia and their relatives consider knowledge and experience of hospital staff when caring for persons with dementia as well as paying attention to their individual needs as important for a good quality hospital experience [11]. At the same time, health professionals find themselves confronted with many barriers to providing adequate care for persons with dementia in the acute hospital setting. One reason is the task-oriented culture of care mainly targeting clinical routine and physical health [12]. Acute hospitals focus on acute medical care and persons with dementia can be considered as a disruption to normal routine [13]. Secondly, professionals in the acute care setting express a need for dementia-specific training and education [7]. The mentioned factors can have a negative impact on the hospital stay of persons with dementia. For example, persons with dementia are at high risk for a delirium during the hospital stay [14]. Furthermore, dementia symptoms can aggravate [15] and result in behavioural or psychological symptoms of dementia (BPSD) [9]. In a study conducted in German acute hospitals, at least one BPSD was observed in 76% of 270 patients with dementia [16]. They experience anxiety, worries and a lack of control [9].

Different interventions were developed to address this demanding situation. Most frequent, evaluations of educational programmes for hospital staff are described [17, 18]. Other approaches are the use of specially trained nurses like the “Cognition Champions Programme” [19] or involving volunteers in acute care [20].

A systematic review of interventions for persons with dementia in acute hospitals found, that the reported interventions showed only few effects on patient outcomes and that there is no sufficient evidence to declare most effective interventions [21]. Furthermore, it was stated that standalone interventions like episodes of staff training are not suitable to improve quality of care in a long-term, sustainable way [22]. Promising results show that interventions, focusing on person-centred care could improve care quality for persons with dementia in acute hospitals [23]. Thereby, addressing organisational structures and processes in hospital seem essential to support a sustainable implementation of person-centred care for persons with dementia [24]. Recent research analysing existing interventions, highlights a multicomponent approach as most promising when developing new interventions for persons with dementia in the acute hospital [21, 25].

One multicomponent approach that also addresses organisational structures and processes are special care units for persons with dementia. A systematic review compared special care units to standard care in acute hospitals [26]. Only three studies were suitable for inclusion [27–29]. Non-significant improvements in readmission rates were found and patients of special care units were more likely to be discharged to their own home [26]. Patients on special care units were more often in a positive mood or engaged, but there was no difference regarding the rates of BPSD. The authors conclude that despite first promising results, the current evidence of specialist inpatient dementia units is limited, and further research is needed [26].

Therefore, we developed and implemented an interprofessional, multicomponent intervention of a specialized unit for persons with cognitive impairment in an acute hospital. In a cooperative project following the principles of action research, we elaborated the interventions and outline of the specialized unit together with the interprofessional team of a geriatric acute hospital. The hospital specializes in providing acute care for individuals aged 60 and above, with a predominant focus on those over 80. Given its emphasis on the elderly population, the hospital has a significant proportion of patients with dementia, constituting 13.5% of the patient population in 2019. The specialized unit aimed at improving the care for patients with cognitive impairment, developing staff competencies and generating knowledge [31, 32]. The collaboration partners participated in the project group and in the research preceding the development of the specialized unit. They also became change agents during implementation process. The project group consisted of three project leaders (nursing scientist, geriatrician and advanced practice nurse) and seven project members (nursing scientist, geriatrician, unit manager, registered nurse, therapist, social worker and study nurse). In phase 1 (exploratory phase, March to August 2020), we refined and specified the already roughly existing outline of the specialised unit in workshops with the project group. In phase 2 (action phase, August 2020 to March 2022), we implemented the multicomponent intervention of the specialized unit and refined it during several iterative action research cycles. One action research cycle included four parts: planning, acting, observing, and reflecting [33]. In phase 3 (evaluation phase, April to October 2022), we evaluated the implementation process and the outcomes by means of qualitative and quantitative methods. The design and the course of the overall study are described in more detail in additional file 1.

**Fig. 1:**
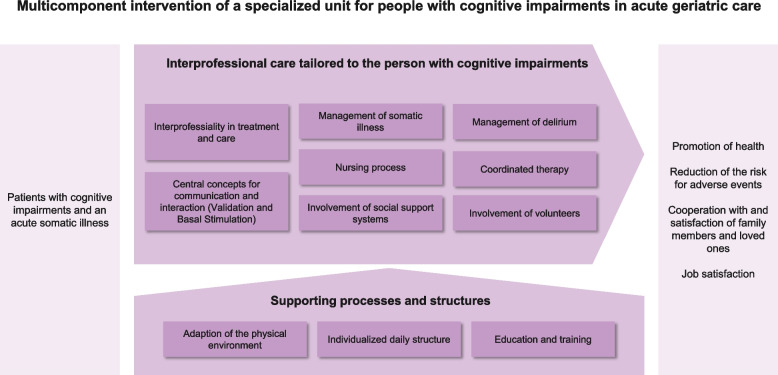
Overview of the multicomponent intervention of the specialized unit for persons with cognitive impairment

The specialized unit encompasses an interprofessional care pathway, staff training, environmental adaptation as well as an individualization of care and treatment. Each component of the multicomponent intervention of the specialized unit comprises a set of interventions. For example, the interprofessional care pathway included a modification of existing communication structures in the interdisciplinary team and an adaptation of the interdisciplinary assessment. Figure 1 summarizes the components of the specialized unit and its interrelations. Additional file 2 comprises more detailed information on the specialised unit.

The implementation of the specialized unit was planned for eight months. However, we had to realize that the implementation did not proceed as anticipated. Although we were a multi-professional project team that was firmly anchored in everyday ward life in lead and executing roles, many components of the interventon took longer and some parts of it were (almost) impossible to implement (e.g. training in the medical team, changes to multi-professional meetings). We extended the action phase in our project and intensified our implementation efforts. After one and a half years, we had to end the action phase and initiate the evaluation phase. Table 1 provides an overview of the implementation status at that time. This did not mean that implementation stopped completely - after the end of the evaluation phase, the clinic continued its implementation efforts outside of the project. In this paper, we only report on the activities within the project.

**Table 1:** Status of implementation of the sets of interventions

**Completely implemented**	**Partially implemented**	**Not yet implemented**
Management of Delirium (Use of the existing multicomponent intervention)	Central care concepts for communication and interaction (Trainings in Validation and Basal Stimulation)	Coordinated therapy Nursing process (Care planning oriented to the person with dementia) Management of somatic illness
Involvement of volunteers (Establishment of 1:1 care when needed) Adaption of the physical environment (Provide employment material on the ward)	Involvement of social support systems (Intensify the flow of information to relatives) Education and training (Aggression management) Individualized daily structure (Individualised mealtimes)	

In the “integrated Promoting Action on Research Implementation in Health Services (i-PARIHS) Framework”, Harvey and Kitson [34] specify successful implementation as a result of agreed project goals and "owning” innovation on the part of engaged, motivated individuals, teams and stakeholders. The four constructs of the i-PARIHS framework are decisive with regard to the success of an implementation project: a) facilitation (the construct that activates implementation), b) innovation (e.g., underlying knowledge sources, clarity, degree of existing practice and values, usability), c) recipients (e.g., motivation, values and beliefs, skills and knowledge, collaboration and teamwork, existing networks), and d) context on a local, organisational and external health system level.

A scoping review about barriers and facilitators to implementing nurse-led interventions in dementia care has identified influencing factors in the following five domains: (1) policy (e.g., financing issues), (2) organization (e.g., organizational culture and vision, resources, management support), (3) intervention/implementation (e.g., complexity of the intervention, degree of clarity of the intervention), (4) staff (e.g., motivation and openness, knowledge and experience), and (5) person with dementia/family (e.g., type and stage of dementia) [35]. Most studies included in this review refer to the nursing home setting. In the acute hospital setting, implementing dementia care interventions in a sustainable way is challenging. Contextual factors as the alignment of ward priorities are particularly important for dementia-friendly interventions in the acute hospital [36]. Thus, the hospital strategy and the task-oriented culture of care [9, 37] focusing on safety and efficiency are considered as superordinate barriers [9]. We had similar experiences in our project: the implementation did not go as planned, which is why we thought to explore possible reasons for this. To ensure a more thoroughly understanding of factors influencing the implementation of complex dementia care interventions in the acute hospital setting, in-depth knowledge about specific facilitators and barriers in this field is necessary.

## Methods

In the context of our project, the current study aims to identify aspects that affect implementation, namely facilitators and barriers to implementing the multicomponent intervention of a specialized unit for persons with cognitive impairment in an acute geriatric hospital.

We conducted a process evaluation as part of a participatory action research study. We examined aspects that affect implementation, as suggested by Moore et al. [38] in their guidance on process evaluation. However, in the guidance the aspects influencing implementation pertain to context. To conduct a more comprehensive examination, we followed the i-PARIHS framework, which views successful implementation as reliant on innovation (intervention), recipients, context, and facilitation [34]. We performed semi-structured individual interviews and focus group interviews with members of the interprofessional team. Additionally, we used data of workshops conducted during the implementation process (phase 2).

### Setting

The study was conducted in a geriatric acute hospital in the German-speaking part of Switzerland. The hospital planned to develop and implement a specialized unit for persons with cognitive impairment and sought therefore scientific collaboration resulting in this action research project. The hospital comprises 90 beds for polymorbid patients over 60 years with functional impairment and an acute somatic illness. The specialized unit consists of 24 beds. The treatment team comprises two senior geriatric assistant physicians (quarterly rotation), xy nursing staff members (unit manager, XY registered nurses, XY other nursing staff members), occupational therapists, physiotherapists and social workers.

### Participants

We conducted fifteen individual interviews with professionals working on the specialized unit. Additionally, two focus groups took place with twelve members of the interprofessional team who worked on other units of the geriatric hospital. To cover different perspectives on the implementation process, we invited members of all professions involved in the treatment team (physicians, nurses, therapists, social workers, volunteers) with different qualifications and roles. We included staff members of (a) the above listed professions, (b) if they had worked at least two years in the geriatric hospital, (b1 – for individual interviews) had worked on the ward were the specialized care unit was implemented for at least six months or (b2 – for focus groups) had worked on another ward for at least six months (persons who assess changes in work processes outside the specialized unit), (c) who spoke German fluently, (d) were able to critically reflect work process, structures and patient as well as staff outcomes, and (e) were willing to share their experiences and assessments during an interview voluntarily. The inclusion criteria applied to individual interviews and focus groups - the participants differed only in the criterion (v - b1 vs. cb). For each focus group, we aimed for 5-8 participants and an interprofessional mix to encourage discussion. The head of nursing development at the geriatric hospital requested them for participation (EZ).

#### *Data* collection

The interview guide was identical for the individual interviews and the focus groups interviews. LA and MK developed the interview guide based on the i-PARIHS framework and the components of the specialized unit. It addressed the following aspects: a) the perceived state of implementation, b) barriers and facilitators to the implementation (with regard to the context, the components of the specialized unit, the project group and the patients), c) changes perceived in one’s own professional team, in other teams and in the interprofessional collaboration, as well as d) recommendations for future projects. AZ and EZ checked the guides for meaningfulness and comprehensibility, and we revised the guides according to their feedback. We conducted an interview with a nurse from the specialized ward to test the guide, which showed that the guide worked well, so we also included the interview in the data corpus. LA and AZ conducted the individual interviews. LA, AZ and NH moderated the focus groups. The interview location depended on the preferences of the participants (office, meeting rooms in the geriatric hospital). We audio recorded all interviews.

For the process evaluation, we also used data collected during the workshops (see additional file 1) of the implementation phase (e.g., workshop protocols, documentation of the observations, milestone plans, and workshop presentations of different project group members).

### Data analysis

We performed a qualitative content analysis following Kuckartz’s content-structuring analysis scheme [39]. This analysis method consists of the following 7 steps: After the initial text work consisting of tagging important segments and writing memos (step 1), we deductively formulated main categories (step 2) for barriers and facilitators according to the i-PARIHS Framework [34]. For this purpose, we used the key constructs of the framework: innovation/intervention, recipient, context and facilitator. We coded all data by means of the initial main categories (step 3). In step 4 we compiled all text segments (codings) with the same main category. Afterwards, we developed subcategories (step 5) following the the i-PARIHS subconstructs and their definitions [40]. We revised the main categories in order to ensure that they are content-specific, unambiguous and expressive. Finally, we coded the entire material by means of the differentiated category system (step 6). Step 7 was initially a category-based evaluation along the main categories, whereby the results for each main category were described in detail and provided with anchor quotations. In addition, correlations between categories were sought and the entire evaluation was transferred to a visualization. The data analysis was carried out by the research team, with two authors developing the initial category system (LA, NZ) and refining and developing it further in several analysis sessions with EZ and AZ. (Interim) results were discussed s in a workshop with the project group and we adapted them in accordance with the consensus findings.

#### Trustworthiness

To enhance credibility, we used purposive sampling to be able to interview persons that were able to provide in-depth information and critically reflect their experiences and assessments. In order to interpret the participants' statements correctly, LA and NZ repeatedly coded parts of the material independently of each other and compared their coding. To ensure that the views of the interview participants were appropriately reflected in the results, we conducted a member check. We organised two meetings during which we presented our findings to four interview participants and asked them about their regarding their agreement with the results and their assessment of the comprehensibility of the results. The translation of the categories and quotations was double-checked by the entire team of authors and a professional translator.

With regard to confirmability and researcher bias, we state that all authors are registered nurses, have experiences in working in acute care settings and in caring for persons with dementia. During the study, LA, MK, and HZ were based in a research institution and lacked direct patient interaction, allowing for an external perspective on processes and structures. NH and EZ, employed at the acute hospital during the study, brought valuable first-hand knowledge but were emotionally more connected to the research subject. To mitigate personal biases, experiences, and emotions, we engaged in team reflections throughout data collection and analysis.

## Results

### Sociodemographic characteristics of the participants

The sociodemographic and professional characteristics of the participants are visible in Table 2.

**Table 2:** Sociodemographic and professional characteristics of the participants

Interview-ID	Profession^1^	Work experience in the health care system (years)	Work experience in the geriatric hospital (years)
I1	Medicine	<30	11-20
I2	Nursing	21-30	21-30
I3	Medicine	11-20	6-10
I4	Social Work	11-20	2-5
I5	Therapy	3-5	2-5
I6	Nursing	11-20	11-20
I7	Nursing	<30	11-20
I8	Nursing	11-20	2-5
I9	Nursing	11-20	2-5
I10	Nursing	6-10	21-30
I11	Volunteers	31-40	6-10
I12	Therapy	21-30	21-30
I13	Nursing	31-40	21-30
I14	Social Work	5-10	2-5
I15	Medicine	31-40	5-10
I16	Nursing	11-20	2-5
FG1_P1	Nursing	<30	2-5
FG1_P2	Nursing	3-5	2-5
FG1_P3	Therapy	6-10	2-5
FG1_P4	Therapy	11-20	2-5
FG1_P5	Nursing	3-5	2-5
FG2_P1	Nursing	21-30	21-30
FG2_P2	Nursing	21-30	2-5
FG2_P3	Nursing	6-10	2-5
FG2_P4	Therapy	11-20	6-10
FG2_P5	Therapy	3-5	2-5
FG2_P6	Nursing	11-20	6-10
FG2_P7	Nursing	6-10	2-5

Interview IDs beginning with "I" indicate participants of individual interviews and interview IDs beginning with "FG" indicate participants of focus groups

### Categories

**Fig. 2:**
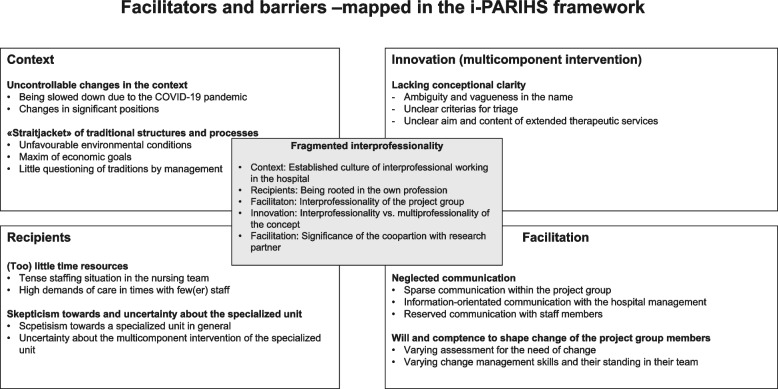
Main and sub-categories according to the i-PARIHS framework

We identified eight main categories depicting inhibiting and facilitating aspects (see Fig. 2). A clear division into barriers and facilitators on the level of main categories was not possible. However, most of the factors describe barriers. Due to multiple obstacles during the implementation process, the introduction of sets of interventions was slowed down or postponed. This had a negative effect on the continuity of the implementation process. Subsequently, it was not possible to implement the multicomponent intervention of the specialized unit completely.

In the following, we describe the main categories and the subcategories in detail.

#### Context: uncontrollable context-related changes

Contextual changes implied numerous interruptions during the implementation process. The participants’ answer to the question: “*What comes to your mind when you think about the implementation process?”* often was “interruption”. From the participants’ point of view, the reasons for interruptions were outside their control.

“*We have already had so many interruptions with a detrimental effect. It's nobody's fault. But now we can no longer afford any interruption […]. Otherwise, everything silts up*.” (Interview I13, pos. 62-64)

Consequences of the COVID-19 pandemic have slowed down the implementation process*,* shifted priorities and created uncertainty. The start of the implementation process was planned for spring 2020 – the time of the first lockdown. Pandemic-related measures resulted in a higher workload, restricted hospital access and changes in patient pathways. Establishing protection/isolation concepts and mastering the increased workload were at the centre of events. For a few months, the specialized unit for persons with cognitive impairment even accommodated patients who had to be isolated due to a COVID-19-infection.

During the implementation process, there was a staff turnover in the hospital management and on the unit (e.g., new CEO, maternity leaves, high fluctuation in the nursing team). Changes in the team structure resulted in a loss of knowledge, project-related continuity and motivation.

#### Context: “Straitjacket” of traditional hospital cultures and structures

According to the interviewees, the hospital culture is characterized by rigid structures and inflexible processes. The participants experienced this as a “straitjacket” severely limiting any development.

The majority of the participants regarded the *environmental conditions* as unfavourable for interventions addressing the needs of patients with cognitive impairment. The spatial structures of the unit resembled the traditional hospital architecture. Due to regulations, changes were impossible. For example, due to the architects' specifications, signage for wayfinding could not be added or adapted, and no pictures or analogue clocks could be hung in the rooms. Additionally, the processes on the unit were designed around the workflow of the interprofessional team. They were highly synchronized and efficiency-orientated. Implementing patient-centred processes oriented towards the needs of persons with dementia (e.g., individualized schedules, extended time frames for care and therapy) was challenging. We were aware of the implementation barrier posed by efficiency-focused processes, but we were taken aback by the remarkable resilience and resistance to change exhibited for these processes. For example, tightly planned schedules made it almost impossible to extend a weekly interprofessional meeting by five minutes.

Furthermore, the participants reported *economic maxims* dominating the allocation of limited resources. The hospital operates as a stock company and as such is profit-oriented. The interviewees mentioned the hospital’s credo to achieve the billing code “geriatric acute care” for each patient. This economic necessity required a certain number of therapeutic sessions within a defined time frame. However, this was difficult to achieve for patients with cognitive impairment. Multiple appointments per day contradicts the needs of this patient group:

“*There was the idea of working flexibly on the third floor. We treat nine patients and, depending on the necessity, one patient a little longer and the other patient for a shorter time. But that doesn't work with this billing system. I'm afraid that this has to be said. It's not feasible if you still want to achieve the billing code geriatric acute rehabilitation for all of them*.” (Interview I6, pos. 46)

The participants experienced the environmental conditions and the economic maxims as inflexible and restrictive. From their point of view, an “individualization” of treatment and care – tailored to the needs of the person with cognitive impairment – is essential for the specialized unit. However, they pointed out that this is hardly possible within the existing organisational culture and the dominating economic requirements. Changing structures and processes (e.g., for interprofessional history taking) was part of the implementation plan. However, it was difficult to achieve due to the firmly entrenched organizational and professional culture.

According to the interviewees, the hospital management’s participation in the implementation process would have been decisive in order to promote structural changes. However, the participants mentioned that even for hospital managers it was difficult to question and to change traditional structures. From the point of view the interviewees, the hospital management behaved in an ambivalent way. On the one hand, they clearly decided in favour of introducing the specialized unit. On the other hand, they did not bring in their authority into the implementation process. Members of the project group also critically reflected that they did not succeed in getting the hospital management “on board”.

#### Recipients: scepticism towards a specialized unit

Professionals working on the specialized unit were sceptical regarding the specialization for patients with cognitive impairment. They also did not know the content of the components of the specialized unit in detail. This caused uncertainty and doubts about whether it would be possible to care for patients with dementia *and* delirium in one unit:

“*I'm basically questioning it. I think we would have to discuss this again in principle. To what extent does it make sense to separate these people? […] Certainly, there are reasons why we should bring them together. But there are also many dangers …*.” (Interview I12, pos. 83).

Many participants expressed their apprehension concerning the burden for the team due to the high intensity of care. Furthermore, the interviewees emphasized that the combination of patients with and without cognitive impairment resulted in different care needs. From their point of view, there is a danger of overseeing the needs of patients without cognitive impairment. In contrast to persons with dementia, they communicate their needs in a way that is less visible and audible. The participants mentioned that they cannot "do justice" to all patients and, therefore, experience an ethical dilemma.

Furthermore, the interviewees also expressed *uncertainties about the components of the specialized unit*. The concrete meaning of “specialization” was unclear to them and they did not know the targeted patient group. In addition, the roles and distribution of tasks in the therapeutic late shift were unclear for the participants. According to them, conception and communication towards the staff were insufficient. Therefore, they felt uncertain and did not implement the interventions:

“*My question is: What is the task for me as a physiotherapist in the late shift? Is it caring for the person? Or is it more a therapeutic activity? In physiotherapeutic terms, I think there is an extremely wide scope.*” (Focus group 1, P3, pos. 22)

#### Recipients: insufficient resources

From the participants’ point of view, caring for patients with cognitive impairment is time-consuming and requires individualized schedules. However, the staffing situation on the specialized unit did not differ from other units. The specialized unit included plans for increased staffing in the nursing team and ongoing staff education. Unfortunately, due to high turnover rates, the enhancement in staff numbers could not be implemented. This prevented the professionals from implementing innovations in general and complicated caring for patients with cognitive impairment in particular. The plan to create additional jobs for nurses proved to be unrealizable, due to lack of applicants for these jobs.

According to the participants, the lack of time resources was the result of the tense staffing situation in the nursing team:

“*I think that the staffing ratio must be high enough. Now it is otherwise ... It is neither fair to the patients nor to the nursing staff. Actually, everyone is overburdened*” (Interview I6, pos. 74).

*A high demand for care* occurred particularly in the late afternoon, in the evening and at night when the staffing level was very low. The participants reported being unable to meet the patients’ needs during these times. This resulted in situations prone to complications:

“*We often have patients with hip transplant. They walk around as if nothing had happened. And that is sometimes a bit difficult for us (laughs).*” (Focus group 1, P4, pos. 50-51)

The COVID-19 pandemic, high demands on nurses and continuing uncertainty, resulted in a high number of resignations. In addition, there were also several maternity leaves. The remaining team struggled to maintain regular patient care. Exceptional patient situations triggered fear and rejection:

“*I find it difficult when we have more than two patients [with an increased need for care]. Then you need more hands to take care of them. When someone constantly calls: 'Hello!' ... And we've really had a lot of [nurses] saying: Maximum one [with an increased need for care]. Due to the burden ... And the young [nurses] say: I don't like it anymore! We had [a patient] who was really so prone to falling ... We had to accompany her everywhere. You couldn't leave her alone for a minute. Otherwise, she left the unit. You reach the limit.*” (Interview I8, pos. 12-13)

During the pandemic, implementation activities were postponed and beds on the unit were blocked. Subsequently, the stress levels in the nursing team were more closely monitored and taken seriously. Stabilization in the nursing team had a higher priority. However, implementation activities were further delayed.

#### Innovation: lacking conceptual clarity

Participants mentioned ambiguities and vagueness concerning the term “specialized unit for patients with cognitive impairment”. The intention was to address patients with dementia *and* delirium. The term “cognitive impairment” should signal that the unit is not exclusively for patients with dementia – it is also open to other patients. However, this complicated the triage. The interviewees reported that the triage should be aligned to the current occupancy situation of the specialized unit. Due to this, it was left to the interpretation of the professionals which patients to treat in the specialized unit:

“*I think it's fair to say that we have a unit for ‘patients with dementia’. I mean, that's not a personal judgement. It's a diagnosis. And now you have to say: We have a ‘unit for patients with cognitive impairment’, I think that is a trivialization! Eighty percent of our people have less than twenty-six points in the Mini Mental State Examination”* (I12, pos. 12).

According to the participants, the aim of the newly introduced “*extended therapy service”* was unclear. Originally, it was planned to make schedules more flexible and to ensure a more even distribution of therapies during the day. For example, therapy sessions for patients with dementia, that could not follow an entire therapy session could be split in two shorter sessions and provided over the course of the entire day. However, the participants reported that they did not know whether therapy should now take place preferably in the late afternoon hours. Additionally, it was not clear, whether this service is focused on care or on therapy.

#### Facilitation: neglected communication

Retrospectively, the participants considered it as crucial to have a viable communication strategy:

“*We never agreed upon the question: How should we inform the persons involved? We should have definitely agreed on that at the beginning: Who informs whom and when? And who is responsible for what?”* (Interview I13, pos. 48)

The participants characterized the communication as unsystematic, sparse and delayed. It can be concluded that the communication measures proved insufficient, despite the team’s already extensive efforts to boost communication through various means, including regular informational events for all employees and ongoing presence in management committees. The initial plan to appoint a dedicated study nurse for the specialized ward to enhance communication among stakeholders and teams fell through due to the inability to fill the position. The communication deficits concerned all persons involved: members of the project group, staff, and hospital management. According to the interviewees, communication within the project group mainly took place in the workshops organized by the researchers. However, the workshops were initially not intended as the central medium of communication. To fulfil this function, more frequent workshops with shorter intervals would have been necessary. The members of the project group recognized the shifted function of the workshop. However, they did not react on it and workshops were held as planned. Outside the workshops, communication was unsystematic, random and spontaneous. The participants mentioned that the project group neglected communication about roles and process design. For example, there was no information about role clarification and about the facilitator role. As a consequence, role interpretations were very inconsistent.

At the beginning, the project group defined the tasks of implementers and co-researchers. However, in the course of the further project, there was no ongoing discussion concerning their tasks. Everyone assumed to know role-associated tasks. No one noted the necessity of increased role clarification. Inconsistencies only became apparent when the implementation was already far advanced. At this point of time, it was impossible to resolve the inconsistencies.

According to the interviewees, communication with the hospital management was primarily information-related. The project leader informed the management on a regular basis about the implementation progress. However, the management was never actively engaged in the project. From the participants΄ point of view, this was a a barrier. Neither specific needs nor responsibilities were addressed.

Communication towards hospital staff was limited to information events. However, these events were seldom and took place rather late. Some respondents were of the opinion that this inhibited implementation. The project “*petered out*” (Interview I1, pos. 11). Interestingly, not all project group members held this view.

Another hindering factor was associated with new team members. There was no strategy for introducing new colleagues to the multicomponent intervention of the specialized unit.

#### Facilitation: motivation and change competence

The motivation to create change and to improve change competence varied among project group members. According to their profession, they had different opinions concerning the *need for change*. Nurses reported that their situation on the unit before the implementation was stressful and frustrating. They were confronted with an overwhelming workload. Therefore, they pushed for change. However, there was no uniform opinion among members of the nursing profession.

The project group members’ *change management skills* varied. This may be caused by different professional *functions* and by their various *standing in their team.* Whereas some project group members had leadership roles, others had no leadership experience at all. Therefore, recognizing the need for action and creating change proved to be difficult for them:

"You need certain leadership skills if you want to share your knowledge with your teams. This is something I will certainly consider in future interprofessional projects. It’s not only about a certain the topic. You also have to think about which people you want to include and what is their standing in the team. I think, we should have asked this question at the beginning.

But in the course of time, certain people were replaced. And then these considerations were no longer in the foreground”. (Interview I16, pos. 30)

Change management skills were relevant for the selection of project group members. However, due to staff turnover, the project group members changed. The successors often did not meet the originally defined selection criteria:

“*Planning such long-term projects is always very difficult. You have consider that there can always be changes – even in key positions. Identifying the right people at the right time – that is extremely difficult. And I think it is also difficult to define a replacement for each function in advance.*” (Interview I16, pos. 60)

#### *Synthesis*^1^*: fragmented interprofessionalism*

According to the participants, treatment and care of persons with cognitive impairment require close cooperation between the professions involved.

On the one hand, the interviewees emphasized that interprofessional collaboration is common in their clinical practice. The already *established interprofessional culture* in clinical practice facilitated the implementation. On the other hand, outside clinical practice, there was limited experience of interprofessional collaboration at the project level. One participant emphasized that this project was the first “*big interprofessional project*” (Interview I16, pos. 12) in the course of many years. It became apparent that the staff was strongly rooted in a monoprofessional culture. The habitus significantly influenced the way in which interprofessionalism was lived. For example, in nursing and in the medical profession, it is customary to cover shifts 24-hours seven days a week. However, therapists were not used to working later than 5 pm. Subsequently, they responded with high resistance to the introduction of a therapeutic late shift.

The professional habitus also influenced the interpretation of one’s role in the project group. Nurses expressed that they felt responsible for the implementation and the steering of implementation activities. They considered it as important to ask questions and to reflect their action. One nurse described the nursing role und thereby the habitus, as "*the mommy for everyone*" (I13, pos. 88). Physicians emphasized the personal responsibility of every individual professional with regard to the implementation:

“*We should implement that officially – we have all the instruments. […] And I would like to proceed pragmatically […]. We all have self-responsibility […]. The roll-out of the individual parts should be left to on one’s own initiative*” (Interview I15, pos. 2)

According to the participants, the *interprofessional composition of the project group* facilitated the implementation. There was one representative of each profession – except for nursing with two clinical representatives and two research representatives. The participants critically reflected the predominance of nursing. Nursing was the profession with the highest contribution of resources, including staff and decision-making-power. On the one hand, this was related to the nurses’ high psychological strain in caring for persons with cognitive impairment. Due to this, the motivation for change was very high on the part of project group members of nursing profession. On the other hand, the majority of the interprofessional team consisted of nurses. The participants mentioned the nursing focus of the multicomponent intervention of the specialized unit and of the implementation:

“*It is still rather too nursing-oriented. […] The initial concept is like this ... Each profession only works side by side. I would like to see togetherness.”* (Interview I14, pos. 106)

The *multicomponent intervention* included some sets of interventions affecting all professions involved. However, other sets addressed only one profession, for instance the management of somatic diseases and the nursing process. Subsequently, the multicomponent intervention unintentionally promoted working *alongside* each other rather than *with* each other.

In addition, entrenchment in one’s own profession became apparent in differences of professional language and concepts. For example, one set of interventions addressed communication with family members. The denomination of this set was widely accepted in nursing but not in the area of social work. Accordingly, social workers did not identify themselves with this set of interventions. Although the project members work together every day, they were not aware of different terminologies.

The participants experienced *the cooperation with researchers* as a facilitating factor. They cherished especially the workshops during the implementation process, introduced by the researchers as part of the action research cycle. In the workshops, they elaborated the sets of intervention in detail, developed implementation strategies and evaluated the ongoing implementation activities.

## Discussion

This process evaluation is focused on barriers and facilitators to implementing a multicomponent, interprofessional multicomponent intervention of a specialized unit for persons with cognitive impairment in an acute geriatric hospital.

Uncontrollable changes like the COVID-19 pandemic and staff turnover in significant management positions slowed down and complicated the implementation. Furthermore, traditional structures and processes obstructed individualised and needs-oriented care. The professionals involved experienced uncertainty concerning the targeted patient group and the triage criteria of the new unit. Furthermore, there was scepticism about the benefits of the specialized unit. The time resources were insufficient. Staff and project group members did not move in the same direction in all phases and aspects of the implementation. Fragmented interprofessionalism was another often-mentioned barrier, since every staff member was rooted in her/his own profession.

The results indicate that the hospital culture was experienced as a significant barrier to implementing the multicomponent intervention of a specialized care unit. A meta-synthesis addressing facilitators of person-centred care for patients with dementia identified the organizational culture and structure as influencing factors in acute hospitals, including leadership support and sufficient time resources for the nurse-patient relationship [24]. In the current project, it was not possible to loosen the ‟straitjacket‟ of traditional hospital structures in favour of a more person-centred care.

This illustrates a limited capacity of the hospital system to promptly address and prioritize the distinctive needs, goals, and experiences of individual persons. Health system responsiveness describes the “experience of people’s interaction with their health system, which confirms or disconfirms their initial expectations” [41]. Responsiveness is high, when health care providers have sufficient resources to dynamically identify the needs of individual people and adapt to them. Standardized approaches across an organization are characteristic for low responsiveness [42]. Bridges et al. [42] point out, that higher complexity of patients’ needs requires a higher level of system responsiveness.

Our results underline this statement. The health professionals stated that “individualization” (i.e. the adaptation of care to the individual and complex needs of the person with cognitive impairment) was a main element of a specialized unit for persons with cognitive impairment. At the same time, hospital processes and structures hindered the professionals to realize individual care. According to the characteristics of system responsiveness, this may suggest that professionals had insufficient skills, autonomy, flexibility and/or resources to adapt to the patients’ needs [42]. In our interviews, the participants did not mention skills and autonomy. However, we can assume that these aspects also influenced the implementation of a more person-centred care. Nurses were confronted with staff shortages in times of higher care needs of persons with cognitive impairment. Furthermore, they found themselves in rigid structures and times frames. This may have prevented the necessary flexibility to ensure needs-orientated care.

Our results suggest that without a fundamental change of the hospital culture, the complete implementation of a specialized unit for persons with cognitive impairment is not possible. A framework for implementing dementia care in acute hospitals reveals that a person-centred hospital culture with person-centred structures and processes has already to be in place. This proves to be the precondition for a successful implementation of optimal dementia care [30].

McCormack and McCance [43] underline the importance of workplace cultures for realizing person-centred care. According to their definition, person-centredness “is enabled by cultures of empowerment that foster continuous approaches to practice development”. According to the “Person-centred Practice Framework”, the quality of practice environment has an impact on the effectiveness of person-centred practice [44]. An enabling practice environment includes supportive organizational systems, appropriate skill mix, shared decision-making systems, effective staff relationships and power sharing [44]. A qualitative study with interdisciplinary teams in acute hospitals shows that care and treatment of persons with dementia in acute hospitals require intensified interprofessional teamwork and communication [8]. Our results confirm the importance of collaborative and effective staff relationships. The participants of our study regarded interprofessional collaboration as “basically good”. However, our results show that the participants were deeply rooted in their own professional cultures. This proved to be a barrier to the implementation process. We were aware of diverging priorities in dementia care among professions and addressed this by developing and implementing the intervention with an interprofessional team. This allowed each profession to contribute its perspective. However, inherent to interprofessional interventions is the fact that they do not completely align with one's own professional culture and habitus. We were surprised at the limited flexibility employees showed in accepting components of the intervention if they did not entirely correspond to their own profession. For example, they employed different terms within their professional jargon, and while they understood each other, their level of engagement in the care process diminished when terminology diverged from their accustomed terms. Also, criteria for prioritizing differed among the professions. They had various foci regarding the care of persons with cognitive impairment. Different cultures of care within a team are described as barriers to implementing dementia care interventions [35]. To support person-centred care, McCance and McCormack [44] emphasize the importance of agreed values, goals and wishes within the interprofessional team. Our results suggest that identifying the values and goals of the team members and negotiating a shared vision is essential before implementing interventions.

In participatory action research, the development of value-based partnerships is considered as a key implementation element [45]. Partnerships should be non-hierarchical as well as based on mutual trust and respect. In our study, the participants were used to work together in clinical daily routine. However, they had no experience in working together in implementation projects. This complicated the implementation, since their ideas of the implementation process differed widely. According to White et al. [45], the clarification of roles and relationships should receive attention in participatory action research – with the aim of taking over new roles and responsibilities. This could be a chance in interprofessional implementation projects in the acute hospital setting. Existing hierarchies and fixed professional roles have to be questioned in order to pave the way for negotiating new roles and responsibilities. Cargo and Mercer [46] describe four partnership stages in an participatory research project: engagement, formalization, mobilization and maintenance. In the second stage (formalization), a mission or vision is established or refined. An organizational structure as well as operation norms are developed or strengthened. Furthermore, Cargo and Mercer [46] point out that sufficient time to develop a partnership and to foster capacity building is necessary. This is a main challenge since formalization of partnerships can consume the first six to twelve months of a project [46].

To consider these aspects in future projects is one of the most important “lessons learned” of our project. The process evaluation shows that partnership activities and role clarification are very important – even if project members know each other well and are already working together in the clinical setting.

Our results add an important factor concerning the stage of formalization in the health care context: In interprofessional project groups, it is important to consider challenges associated with interprofessional structures and habitus. Thus, it takes further activities to clarify and to redefine the profession-specific roles within the project [47].

There are various strengths and limitations related to the process evaluation reported in this article. A strength of this process evaluation was its integration into the overarching action research design of the study. It allowed to perform research *with* people instead of *on* people. The inclusion of clinical project group members as co-researchers enhanced participation during the research process. Power, focus of questioning as well as interpretation of the analysis were shared [45]. The strong embedding of the co-researchers in the research field facilitated the recruitment of interview partners. They knew exactly who had the best information and who was suitable for engaging in self-critical reflection which facilitated our purposive sampling. The familiarity, that promoted mutual trust, encouraged the interviewees to speak very openly about their experiences and assessments. It also became apparent that the joint interpretation of the data with the clinical co-researchers shed a different light on the results and changed the embedding of statements. By bringing in the scientific perspective, it was increasingly possible to embed the statements theoretically, which also provided points of reference for further work in the hospital. This resulted in a very deep understanding of the barriers and facilitating factors in the implementation of the multicomponent intervention. We regard the facilitated bidirectional education between researchers and co-researchers as one of the main benefits of the participatory action research design . Furthermore, the high identification with the results and their specificity for the hospital increased their use for further development.

However, relationship building may also have had a negative side. The researchers knew the participants and were familiar with the implementation process. Due to this, the participants did not elaborate on every detail. They assumed that the researchers “knew what they mean”. Therefore, the researchers often did not ask for further details. They deliberately refrained from too insistent questions that may have caused irritation and jeopardized trust.

During the evaluation process, it was difficult for the researchers to distance themselves from the field. It took some time, intense analysis of the interview data and reflective conversations in the research team in order to maintain an objective researcher role.

For practical reasons, we decided to base our questions on the key constructs of the i-PARIHS framework. However, we do not have data concerning all i-PARIHS-sub-elements [48]. For example, in the key construct ”innovation” we only found information on the sub-element ”clarity”. Barriers concerning other sub-elements may have remained undiscovered. To receive information on all sub-elements of the PARIHS-framework, a complete integration of PARIHS-sub-elements into the interview-guide would have been necessary. The omission of the PARIHS-sub-elements resulted in missing information, for example concerning recipients’ skills.

Another limitation of our study refers to the short-term validity of the results. Fundamental organizational changes are currently taking place in the geriatric hospital. Independent of these organizational changes, the results of our study can provide key information for future developments and for other implementation projects in similar contexts.

## Conclusions

Our results provide in-depth information about facilitators and barriers to implementing a specialized unit for persons with cognitive impairment in an acute hospital. Strategically planned, precise and detailed communication proved to be essential to facilitate the implementation. To avoid scepticism and rejection during the implementation process, it is necessary to be familiar with the organizational culture and the specific work cultures of the professions involved. The implementation of many sets of interventions requires an organizational development process. There is a risk of undervaluing resources and time requirements associated with organizational development. Creating innovative workplace cultures and developing team competencies in times of consistent changes in the organisation takes time. Providers conducting implementation projects in the field of dementia care and delirum in the acute hospital setting may benefit from organizational and practice development approaches. Although such approaches are time-consuming, they contribute to a de-implementation of traditional patterns and to the development of a person-centred culture.

### Supplementary Information

**Additional file 1.** An overview of the design and course of the overall project.

**Additional file 2.** The specialized care unit for persons with cognitive impairment: An overview of the multicomponent intervention of and its implementation strategies.

## Data Availability

The datasets generated and analysed during the current study are not publicly available for reasons of protecting the anonymity of the participants. For plausible reasons, parts of the data are available from the corresponding author on request.

## Abbreviations

i-PARIHS: “integrated Promoting Action on Research Implementation in Health Services (i-PARIHS) Framework

## Footnotes

1 The category “Fragmented interprofessionalism” includes sub-categories of all four i-PARIHS-key constructs (context, recipients, innovation, facilitation).
