# Supplementary material for: Facilitators and barriers to implementing a specialized care unit for persons with cognitive impairment in an acute geriatric hospital: a process evaluation
Source: BMC Geriatr. 2024 Jan 6;24:29. doi: 10.1186/s12877-023-04612-8 (PMC10771665; doi:10.1186/s12877-023-04612-8)
Supplement: Supplementary file 1 — Additional file 1. An overview of the design and course of the overall project. [file 12877_2023_4612_MOESM1_ESM.pdf]

Additional file 1:
An overview of the design and course of the overall project

We conducted a practice development project that was carried out using participatory action research approach in three phases [1]. As the multicomponent intervention of the specialized unit is rather complex, we expected dynamic development processes. The action research approach allowed us to address these, to explore them scientifically and to stimulate systematic reflection processes. In accordance with the participatory action research, the project was planned and carried out together with the people involved, whose social world and meaningful actions were investigated. By combining the knowledge interests and perspectives of practice and science, we pursued the two objectives: understanding and changing [2]. The action research approach is divided into the three phases of exploration, action, evaluation.

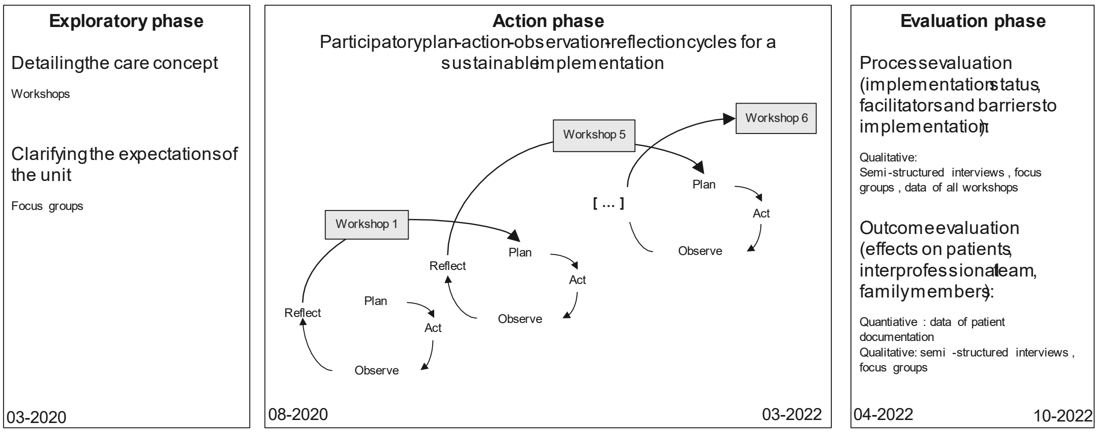

Figure 1: Design and course of the study

Participation in the course of the study: co-researchers and project group

In participatory action research, stakeholders are active participants throughout the entire research process, assuming the role of co-researchers. Participation, defined as involvement with decision-making authority, spans from establishing objectives to disseminating results. The overall project was coordinated and executed by the co-researchers. Throughout the exploration (development) and action phases (implementation), they received support from the interprofessional project group.

The co-researcher: In our specific case, participation translated into shared project management between the hospital (geriatrician and advanced practice nurse) and research partner (nursing scientist). Co-researchers comprised employees from both institutions, including the three research assistants from research facility and an advanced practice nurse from the hospital as well as the project managers.

The project group: To ensure the creation of a comprehensive and well-executed concept, active involvement of all professions engaged in the treatment and care process was essential. Consequently, an interprofessional project group was formed, consisting of representatives from the professions working on the 3rd floor who played a significant role in implementing the concept (refer to Table 4). The co-researchers were also integral to this group. The project group actively participated in the development and implementation of the

multicomponent intervention, possessing decision-making authority. It played a key role in defining sets of interventions, determining their content, and overseeing the implementation process. Representatives from each profession within the group contributed their unique perspectives on the treatment and care process, along with their specific needs. The nursing scientists from the research facility added an external and scientific perspective to the collaborative efforts.

Exploration phase (phase 1)

In the exploration phase (phase 1) we examined in-depth the outline and multicomponent intervention of the specialized unit for persons with cognitive impairments, its characteristics, the expected changes, and the planned implementation strategiesThe project team jointly defined these central aspects of the multicomponent intervetnion in two workshops. In the first workshop, we developed the basis for a logic model [3], which links the sets of interventions of the component with the expected changes. The scientists of the project group elaborated the first draft of the logic model, which then was validated in a second consensus workshop by the project group. Furthermore, we conducted two focus groups with members of the interprofessional team of the geriatric hospital to enquire their expectations of the specialized unit. The results of the workshops and the focus groups built the basis for the first reflection in the action phase.

Action phase (phase 2)

The action phase (phase 2) encompassed participatory action reflection processes to further develop and sustainably implement the multicomponent intervention of the specialized unit for persons with cognitive impairments. For this purpose, we carried out six action research cycles with the phases planning - action - observation - reflection [4], within which we carried out the planning and reflection in workshops with the project groups and worked individually between these workshops.

- Reflection – Planning (Workshops): We reflected the implementation status, the selected strategies and influencing aspects. The implementation status of previously planned interventions was assessed and if necessary adaptions to the implementation strategies or the content or format of the interventions determined. In addition, one to two sets of interventions were planned. These included the operationalization of the interventions, determination of implementation strategies, planning of activities including time and personal resources as well as the definition of responsibilities. Each task was assigned to at least one project group member. The workshops were moderated by two nursing scientists and all project group members participated. The workshops were documented in a written protocol, a photo protocol of flipcharts and other presentation material, as well as transcribed audio-records.
- Action: The defined sets of interventions were implemented following the defined implementation strategy.
- Observation: The project group conducted observations and documented them in specific protocols and in the milestone planning, categorizing them under "implementation status" and "comments."

The evaluation phase (phase 3)

The evaluation (phase 3) included a process and an outcome-evaluation.

In the process evaluation we addressed three research interests: a) the implementation status, b) the implementation strategies and c) barriers and facilitators to implementation. In this paper we report on the barriers and facilitators. The implementation status is briefly described in the background of the paper and the implementation strategies in additional file 2. The methods employed for process evaluation were consistent across all three research interests and are described in the methods section of this paper. We posit that in the data analysis of the implementation strategies the "Refined Compilation of Implementation Strategies" of the ERIC project [5] served as a deductive guide in our data analysis of implementation strategies and was supplemented inductively based on the gathered data.

The outcome evaluation dealt with the impact of the specialized unit on a) patients with cognitive impairment, b) the interprofessional team and c) family members and loved ones. To (a) investigate effects on patients we compared routine data of patients with cognitive impairments before the specialized unit was implemented with routine data of patients after speczialized unitt was implemented by means of descriptive and inferential statistics. To (b) explore changes experienced by the interprofessional team we conducted semi-structured interviews and focus groups with the team members. (c) We also interviewed family members and loved ones to study their experiences on the specialized unit. All qualitative data was analyzed with Kuckartz's content analysis [6].

References

1. Meyer J. Qualitative research in health care. Using qualitative methods in health related action research. BMJ. 2000;320:178–81. doi:10.1136/bmj.320.7228.178.
2. Bergold J, Thomas S. Partizipative Forschungsmethoden: Ein methodischer Ansatz in Bewegung 2012. doi:10.17169/FQS-13.1.1801.
3. W.K. Kellogg Foundation. Logic Model Development Guide: Using Logic Models to Bring Together Planning, Evaluation, and Action. 2004. https://www.bttop.org/sites/default/files/public/W.K.%20Kellogg%20LogicModel.pdf. Accessed 4 Aug 2020.
4. Kemmis S, McTaggart R. Participatory Action Research-Communicative action and the public sphere. In: Denzin N, Lincoln Y, editors. Strategies of Qualitative Inquiry. Thousand Oaks: Sage; 2007. p. 271–330.
5. Powell BJ, Waltz TJ, Chinman MJ, Damschroder LJ, Smith JL, Matthieu MM, et al. A refined compilation of implementation strategies: results from the Expert Recommendations for Implementing Change (ERIC) project. Implement Sci. 2015;10:21. doi:10.1186/s13012-015-0209-1.
6. Kuckartz U. Qualitative Inhaltsanalyse. Methoden, Praxis, Computerunterstützung. 4th ed. Weinheim, Basel: Beltz Juventa; 2018.
