# Supplementary material for: Facilitators and barriers to implementing a specialized care unit for persons with cognitive impairment in an acute geriatric hospital: a process evaluation
Source: BMC Geriatr. 2024 Jan 6;24:29. doi: 10.1186/s12877-023-04612-8 (PMC10771665; doi:10.1186/s12877-023-04612-8)
Supplement: Supplementary file 2 — Additional file 2. The specialized care unit for persons with cognitive impairment: An overview of the multicomponent intervention of and its implementation strategies. [file 12877_2023_4612_MOESM2_ESM.pdf]

Additional file 2:
The specialized care unit for persons with cognitive impairment: An overview of the multicomponent intervention of and its implementation strategies.

The multicomponent intervention of the specialized unit for persons with cognitiv impairment

Based on a Action research approach, an interprofessional working group developed the multicomponent intervention of the specialized unit in the exploration phase, refined it in the action phase and revised it based on observations and experiences. (more information on the methodological procedure can be found in additional file 1).

The following diagram provides an overview of the components of the intervention (centre), the target group (left) and the objectives (right).

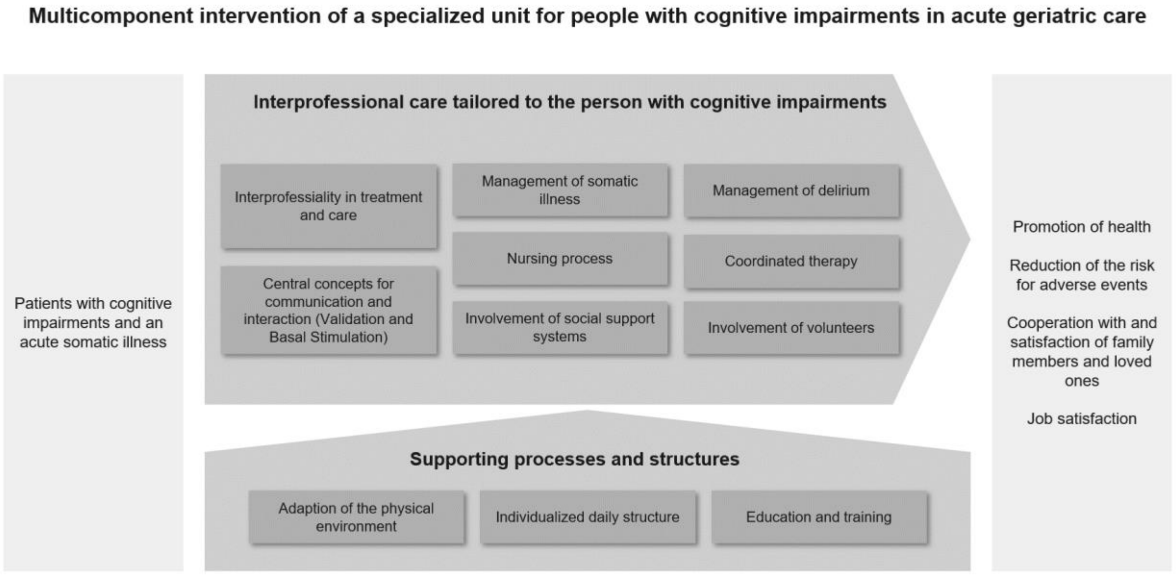

Figure 1: Multicomponent intervention of the specialized unit for people with cognitive impairments

The fundamental principle or guiding belief of the specialized unit is to actively consider the needs, preferences, goals, and resources of individuals with cognitive impairments on an equal basis with patients and their families. The treatment and care is interprofessional. The team, composed of doctors, nurses with varying levels of training, therapists, and social workers, collaborates extensively.

The individual components of the specialized unit are explained below.

Interprofessionalism in treatment and care

The foundation of the treatment and care pathway, as outlined in the internal document "Geriatric Treatment". To be able to care for persons with cognitive impairment a greater emphasis on interprofessional collaboration is necessary. In this regard, adjustments were made to existing communication and information structures, by extending meetings, reducing

redundancies and implementing new information structures specific to cognitive impairment. Systematic access to the patient file (providing information on behavioral problems and psychosocial stress of relatives) in the previous institution was established and the collection of medical history was expanded (now including topics such as preferences in occupation, mobility, sleeping behavior, family history). Moreover, redundancies in the medical history data collection process were eliminated. The existing communication structure "Interprofessional Treatment Meeting" was modified. The interprofessional team convenes weekly to formulate treatment plans and goals, taking into account physical, cognitive, and psychological aspects. This meeting was extended by a comprehensive review of complex cases of persons with cognitive impairments. Each week at least one case review can be addressed. Other established interprofessional communication structures contribute to the organization of the care and treatment pathway. Yet, the majority of collaboration occurs through one-on-one communication. Efforts have been made to foster closer ties within the interprofessional team, facilitating swift problem-solving.

Central concepts in communication and interaction (Validation and Basal Stimulation)

The concepts of "Validation" and "Basal Stimulation" form the central basis for care and communication on the specialized unit for persons with cognitive impairments.

Validation is a communication method that enables access to the world of experience of persons with dementia. The focus is not on the content of the conversation, but on the experience of being taken seriously in one's own feelings, impulses and perceptions. [1] Basal Stimulation is a concept for supporting people for whom the ability to communicate via perception is central. Basal Stimulation enables olfactory, tactile, auditory, visual and gustatory stimuli to be used to support a holistic perception [2]. Both concepts were already used by some nursing staff members and occupational therapists. As part of the multicomponent intervention all staff members of the specialized unit should undergo at least one training in both concepts to enable them to use the concepts in their daily interactions with persons with cognitive impairments. To promote the application of acquired knowledge, bedside training sessions conducted by qualified trainers are offered.

Management of somatic diseases

The management of somatic diseases adheres to geriatric treatment principles, guided by the biopsychosocial model of health and illness. As there is a heightened risk of over- or under-treatment for patients with cognitive impairments, discretionary decisions are often required, considering the preferences of those affected and their relatives in a process of shared decision-making.

Nursing process

The nursing process on the specialized ward is fundamentally similar to other units, with an expanded medical and social history content and tailored nursing interventions specific to the patient group.

Involvement of the social support system

Due to cognitive impairment, engaging social support (such as family members, loved ones, and formal caregivers) becomes crucial. For social workers, this entails providing additional psychosocial counseling for relatives during the hospital stay. Given that this patient group often transitions to nursing homes or more complex home care settings, addressing financial and social law matters becomes significantly important. For this reason, social workers are in contact with patients and their relatives from the moment they are admitted to the specialized unit in order to plan their discharge.

In addition, relatives are also involved during the hospital stay to support nursing and care of the patients if necessary. In addition to extended visiting arrangements, there is also the option of rooming-in.

Delirium management

Part of the multicomponent intervention is a delirium guideline. The focus is on prevention, early detection and early treatment.

Coordinated therapy

The hospital offers physiotherapy, occupational therapy, speech therapy and nutritional therapy. For people with cognitive impairments, the focus is on recognizing, promoting and maintaining cognitive, psychosocial and motor resources. Treatment sessions are typically provided in 30-minute intervalls, with the recommended completion of ten sessions within a 7-day timeframe. To address the challenges faced by patients with dementia, who may struggle to perform optimally at scheduled times or adhere to full sessions, therapy schedules have been tailored to individual needs. Additionally, a therapeutic late-shift has been introduced to extend therapeutic support into the early evening, enhancing accessibility for patients.

Engagement of volunteers

Volunteers play a crucial role in the care team on the specialized ward, contributing in two main ways. Firstly, they offer one-on-one care for patients exhibiting challenging behavior. Secondly, they provide an afternoon service with various occupational activities for all patients. Recognizing the demanding nature of their role, nursing staff regularly check on volunteers, offering stress-relieving activities as needed.

Supporting processes and structures

Environmental design

Due to architectural specifications, the design of the station could not be changed. Therefore, measures were taken to support orientation in the dimensions of time, place and person (clocks, photos, door labels). In addition, the lounge was redesigned. It was furnished in such a way that it looks homely and inviting and offers opportunities for a wide range of activities and social interaction.

Individualized daily structure

Care on the specialized ward requires a daily structure that is individually adapted to the patient. For this reason, nursing tasks were carried out in accordance with the patient's usual daily routine - as far as possible.

Training and further education

The hospital provides ongoing education and training for its employees. Specific further training courses for the specialized unit were defined and integrated in the hospital wide training schedule and platform. Amongst these were introductory courses in dementia and delirium, trainings in the central concepts Validation and Basal Stimulation, bed side trainings in these concepts, trainings in aggression management and de-escalation as well as case reviews.

Implementation strategies

Among other things, the process evaluation served to describe the implementation strategies used. The methods for data collection and analysis are described in additional file 1 – here the results are presented.

The results of the implementation strategies are based on the taxonomy of the "Refined Compilation of Implementation Strategies" of the ERIC project [3]. However, not all implementation strategies can be categorized in this taxonomy. All applied strategies are shown in Table 1.

Table 1: Implementation strategies and their concrete realization in the project

Domain of the implementation strategy	Part of the ERIC taxonomy	Implementation strategy	Realization of the strategy in the project
Communication & involvement of stakeholders	Yes	Form a coalition	A wide variety of coalitions were formed for the specialized ward (project group, connection of the project group to the voluntary service and other important key positions in the hospital [ward management; bed scheduling] and in the neighbouring hospital [referring offices]).
Communication & involvement of stakeholders	Yes	Developing academic partnerships	The project is based on an Academic Practice Partnership (joint utilization of research and practical skills in implementation).
Communication & involvement of stakeholders	Yes	Involve hospital management	The hospital management initiated and commissioned the project. They were informed at regular intervals at formalized meetings about the status of implementation, barriers and adaptations. However, there was only limited success in involving them and making them "participants".
Communication & involvement of stakeholders	Yes	Obtain formal commitment	The formal commitment of the hospital management was obtained during the presentation of the interim status. The project group worked with the hospital management to define urgent activities, appoint responsible parties and set the respective time frames. The result was recorded in writing.
Communication & involvement of stakeholders	No	Regular information about the innovation, the implementation status and the next steps	The hospital management, the staff on the specialized ward and on the other units were regularly informed about the specialized unit, its implementation status and the next steps. The frequency, content and level of detail of the information was tailored to the presumed needs of the target group. The hospital management was informed by the operational project management, the employees by the project group members of their own profession.
Communication & involvement of stakeholders	No	Informal exchange	The project group members exchanged information informally outside of the workshops. During informal meetings on the ward, implementation strategies, interventions etc. were discussed and next steps were determined.<br>As the project group members were anchored in the interprofessional team on the specialized ward, the information and training of individual employees was unsystematic and unstructured.
Facilitation	Yes	Involve facilitators	The members of the project group acted as facilitators. Together, they drove the implementation forward, jointly recognized areas in need of change and developed and implemented adaptation options.
Facilitation<br><br>Communication & involvement of stakeholders	No	Definition of internal, operational project management	The internal project management took over the operational management and organization during implementation. It was the point of contact for all members of the project group, employees and the hospital management. They had decision-making responsibility and managed the implementation.
Facilitation<br><br>Communication & involvement of stakeholders	Yes	Organize meetings of the clinical implementation team	The clinical implementation team consisted of the project group, which met for workshops during the exploration and action phases. The time frame was defined. However, it was not always possible for all members to be present for the entire duration of the workshop. Meetings beyond the workshops

Domain of the implementation strategy	Part of the ERIC taxonomy	Implementation strategy	Realization of the strategy in the project
			took place depending on the upcoming activities from the milestone planning and were planned individually.
Facilitation	Yes	Identification and preparation of champions	This was planned in the nursing department. Team members were to be defined as topic owners for individual components of the intervention and prepared for their role. Due to the high staff turnover, this strategy was not successful. However, there are plans to define topic owners as soon as the situation in the team stabilizes.
Needs analysis	Yes<br>Yes	Local needs analysis<br><br>Assessment of willingness to perform and identification of obstacles	Part of the reflection processes in the workshops of the action phase
Project planning	Yes	Modelling and simulation of change	In the exploration workshops, the project group modelled the multicomponent intervention of the specialized unit by transferring the sets of intervention and their effects into a logic model. This also includes outcomes and thus shows the expected changes.
	Yes	Develop a formal implementation blueprint	Objectives and interventions are defined in the components of the specialized unit. The written description of the multicomponent intervention and the milestone planning form a kind of "blueprint".
Evaluative-responsive approach	Yes	Consciously review the implementation	This took place through reflection in the workshops, during which the milestone planning was discussed and the implementation of the activities was reviewed. In addition, individual interventions were reviewed by the members of the project group - either regularly or on an ad hoc basis. One example of this is the implementation of extended and external anamnesis - here, project group members regularly checked whether the employees were recording them for patients with cognitive impairments and how they were recording them.
Evaluative-responsive approach	Yes	Perform small tests cyclically	During the project, individual activities were initially tested on a small scale, piloted so to speak. One example is the cyclical testing and successful roll-out of childcare services for the voluntary service at fixed times.
Evaluative-responsive approach	No	Plan iterations for evaluation and adaptations during implementation	Milestone planning allows the implementation status to be reviewed, further interventions to be introduced and adaptations to be made. The cyclical approach made it possible to adapt interventions that were not working and introduce a modified version.
Evaluative-responsive approach	No	Align implementation activities with the resilience of employees	Due to uncontrollable contextual changes, the employees on the specialized ward, especially the nursing staff, were exposed to high levels of stress. This changed the resilience of the team. The project group recognized this and reacted by reducing the scope and speed at which new interventions were implemented. For example, the given resilience was taken into account during triage and not all patients potentially suitable for the specialized ward were transferred to it.
Knowledge transfer	Yes	Create a collaborative learning environment	In the workshops of the action phase, the project group members created a collaborative learning environment through joint reflection processes.
Knowledge transfer	Yes	Carry out ongoing training	Training courses were offered on certain parts of the multicomponent intervention, e.g. on central concepts. Some are already part of the training concept and are offered at regular intervals. In addition, project group members (sometimes together with external trainers) developed new training courses, which are also offered on a recurring basis. Furthermore, the specialized unit and its components were integrated into the training of new employees, which also takes place on a regular basis.
Knowledge transfer<br>Kommunikation	Yes	Conduct educational meetings	Employees were each informed about the specialized unit, specific content of the compenents or the implementation of a specific activity by project group members from their profession at meetings, trained in this or current challenges in implementation were analyzed. Team meetings were used for this purpose, but also individually scheduled briefings, which serve to educate a specific employee.
Knowledge transfer	Yes	Use train-the-trainer strategies	The plan is to train everyone in the key concepts of validation and basal stimulation. As this is not possible immediately, some

Domain of the implementation strategy	Part of the ERIC taxonomy	Implementation strategy	Realization of the strategy in the project
			employees will be trained first, who in turn will be deployed as multipliers on the ward.
Knowledge transfer	Yes	Identification and preparation of champions	In the team of carers, topic owners were to be defined for individual sets of interventions and prepared for their role. Due to the high staff turnover, this strategy has not been successful to date. However, it is planned to define topic owners as soon as the situation in the team has stabilized.
Adaption oft he context	Yes	Change documentation system	In order to implement the changes to the medical history in everyday life, the documentation system had to be changed. Here it became apparent that the project group members were not sufficiently aware of what changes could be made to the documentation system and how time-consuming the changes would be. While the introduction of an interprofessional anamnesis failed, the integration of the new data sheets for the external anamnesis, the extended anamnesis and the visualization of these in the documentation of the anamnesis for all professional groups entailed relatively little effort.
Adaption oft he context	Yes	Facilitating the transfer of clinical data to providers/other organizations	This was carried out in the project to implement several sets of intervention: In collaboration with all relevant people at the interfaces, the project group changed the flow of information between the cantonal hospital, as the primary referrer, and the GK. As a result, more information is now available and it is also much more up-to-date. In addition, a subgroup of the project group revised the information flow of clinical data from the CC to the subsequent service providers with regard to content, systematization and topicality. To this end, they also suggested a standardized training and transfer management system in the field of nursing at cantonal level and implemented this in collaboration with the core group for nursing development in the canton of St. Gallen.
Adaption oft he context	Yes	Change physical structures and equipment	A subgroup of the project group evaluated the existing physical environment and adapted it as part of the multicomponent intervention (supporting structures and processes). To do this, they broke down the components into individual activities. Two members of the project group were primarily responsible for their implementation. In particular, architectural specifications, budget and their input periods had to be taken into account.
Adaption oft he context	yes	Creating new clinical teams	This was planned - a new nursing team was to be put together from the hospital's existing staff for the specialized ward. This was not possible due to the low level of interest shown by staff in working on the specialized ward and the limited staff resources.

References

1. Brüggemann J, Brucker U, Eben Eea. Grundsatzstellungnahme. Pflege und Betreuung von Menschen mit Demenz in stationären Einrichtungen. Essen; 2009.
2. Bienstein C, Fröhlich A. Basale Stimulation® in der Pflege: Die Grundlagen. 8th ed. Bern: Hogrefe AG; 2016.
3. Powell BJ, Waltz TJ, Chinman MJ, Damschroder LJ, Smith JL, Matthieu MM, et al. A refined compilation of implementation strategies: results from the Expert Recommendations for Implementing Change (ERIC) project. Implement Sci. 2015;10:21. doi:10.1186/s13012-015-0209-1.
